# Cross-sectional study for determining the prevalence of Q fever in small ruminants and humans at El Minya Governorate, Egypt

**DOI:** 10.1186/s13104-017-2868-2

**Published:** 2017-10-30

**Authors:** Mostafa F. N. Abushahba, Abdelbaset E. Abdelbaset, Mohamed S. Rawy, Sylvia O. Ahmed

**Affiliations:** 1 Department of Animal Hygiene & Zoonoses, Faculty of Veterinary Medicine, Assiut University, Asyut, 71526 Egypt; 2 Clinical Laboratory Diagnosis, Department of Animal Medicine, Faculty of Veterinary Medicine, Assiut University, Asyut, Egypt; 3 Department of Theriogenology, Faculty of Veterinary Medicine, Minia University, El Minya, Egypt

**Keywords:** Q fever, Zoonosis, Small ruminant, Abortion, Seroprevalence, Egypt

## Abstract

**Objective:**

Q fever is a febrile illness caused by the bacterial pathogen *Coxiella burnetii* (*C. burnetii*) and is transmitted to humans from small ruminants via contaminated secreta and excreta of infected animals. This pathogen threatens public health; however, little is known regarding Q fever prevalence in humans and small ruminants. Therefore, we employed a cross-sectional design to determine the Q fever seroprevalence and the associated risk factors in small ruminants and their owners in El Minya Governorate, Egypt between August 2016 and January 2017.

**Results:**

The seroprevalence of *C. burnetii IgG* antibodies was 25.68% (28 of 109), 28.20% (11 of 39) and 25.71% (9 of 35) in sheep, goats, and humans, respectively. None of the studied variables in small ruminants differed significantly between the seropositive and seronegative animals. There was a significantly higher prevalence (*P* = 0.0435) and increased odds of exposure was also observed among women (odds ratio, OR = 5.43 (95% CI 1.058–27.84) when compared to men; nevertheless, no significant difference was noted between the infection rate in small ruminants and humans. This study clearly points out that Q fever may be emerging in the area which lay the foundation for early prediction and better management of possible future outbreaks.

**Electronic supplementary material:**

The online version of this article (10.1186/s13104-017-2868-2) contains supplementary material, which is available to authorized users.

## Introduction

Q “query” fever was primarily used to depict the inexplicable febrile illness that occurred among abattoir workers in Australia in 1935. Its causal agent remained enigmatic for a brief period, then was identified and named *C. burnetii* [1]. Based on recent phylogenetic studies, this agent was found to be closely related to *Legionella* and no longer regarded as Rickettsia [2]. In addition to man, *C. burnetii* is adapted to several animal species primarily including sheep and goats. In these primary reservoirs, the disease is mostly dormant; however, *C. burnetii*-induced abortions can occur in clinical cases, with massive bacterial shedding in placental membranes, birth fluids, milk, and feces in both conditions [3]. Once humans are exposed (through inhalation, contact or ingestion), Q fever remains dormant in most of the infected cases whilst clinical infections are expressed either in acute (self-limited febrile illness, pneumonia or hepatitis) or chronic forms (principally endocarditis) [1]. The various non-specific clinical manifestations associated with *C. burnetii* infection hampers prompt diagnosis, resulting in the development of chronic form as well as massive disease underestimation [1, 4]. Long ago, the disease has been considered an emerging public health concern in many countries [5–8]. Despite Q fever substantial worldwide potential, several localities in Egypt still either neglected or have scarce epidemiological information about the disease, such as El Minya Governorate. Therefore, this work aimed to determine the preliminary seroprevalence of Q fever in El Minya and the associated risk factors in small ruminants and their owners.

## Main text

### Methods

#### Sampling area

**Fig. 1:**
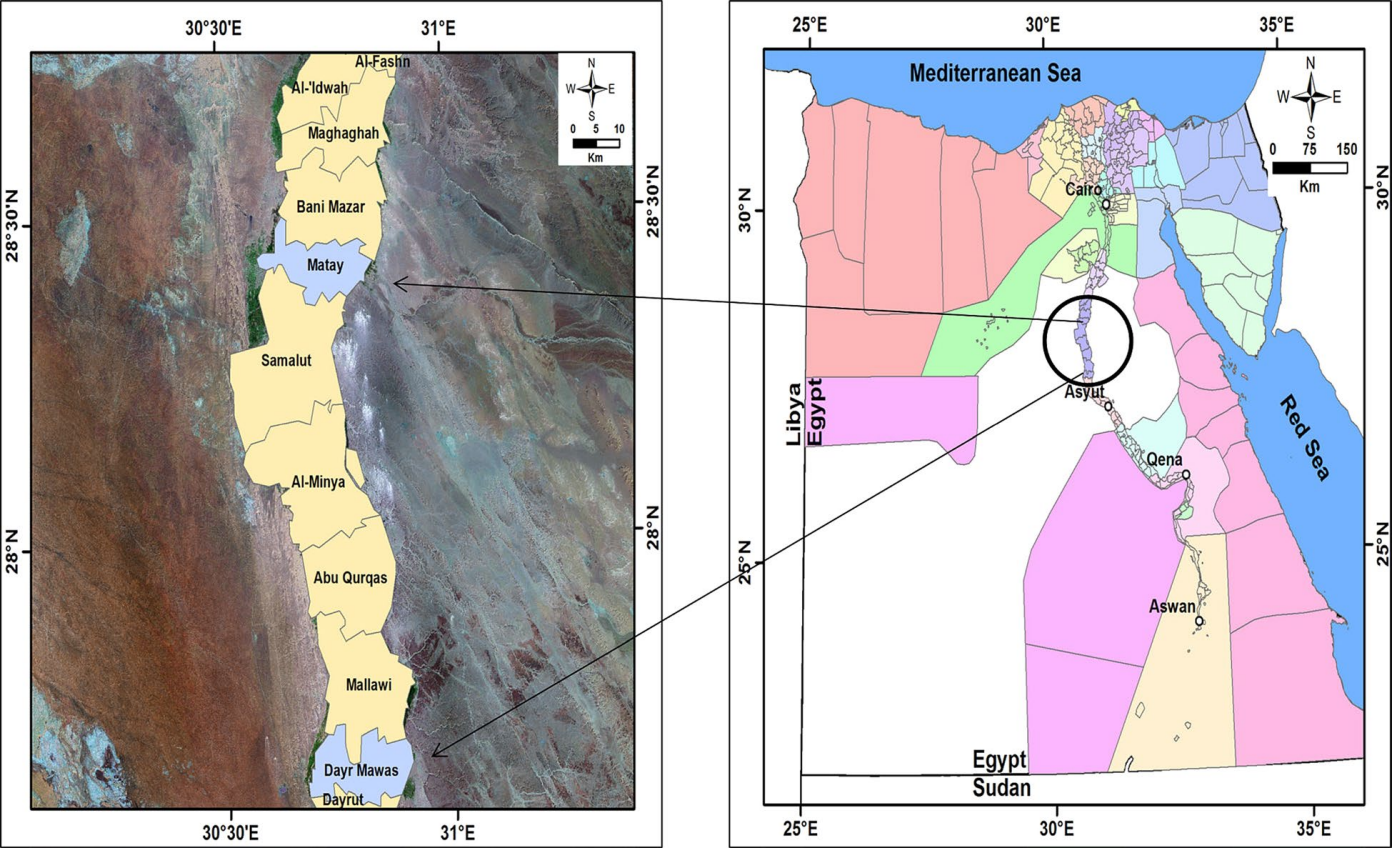
Map showing the sampling area with the survey sites of Q fever. El Minya Governorate is highlighted by a circle on the map of Egypt. Samples were collected from different villages located at Dayr Mawas and Matay districts and are shown by a blue color in the map of the governorate (Downloaded from http://www.gadm.org and modified by ArcGis software)

The present study has focused on El Minya Governorate (~ 245 km south of Cairo) to clarify the preliminary epidemiological status of Q fever (Fig. 1). The majority of El Minya residents live in rural areas and mainly depend on agriculture. They rear sheep and goats on a small-scale (2–50 animals) either separately or in one herd, as a source of financial security, meat, wool, or rarely milk. The area was selected because of no epidemiological surveys regarding the disease has been conducted before.

#### Animal sera

A total of 148 serum samples were randomly collected from 109 sheep (females) and 39 goats (7 males and 32 females) from different villages at Dayr Mawas and Matay districts. The mean age of the selected animals was 4.31 ± 1.78 and 3.66 ± 1.67 for sheep and goats, respectively. Blood samples were collected by jugular venipuncture using sterile syringes and injected directly into plain vacutainer tubes. Labeling numbers and the respective data, such as locality, age, gender, pregnancy status, and abortion history were included. The tubes were kept in an ice box and transferred immediately to the laboratory at the Department of Animal Hygiene & Zoonoses, Faculty of Veterinary Medicine, Assiut University. The collected blood samples were centrifuged at 1800×*g* for 15 min and the sera were harvested and stored at − 20 °C until analyzed.

#### Human sera

A sum of 35 individuals (mean age 38.63 ± 11.70) including 22 males and 13 females were included where all were either sheep and/or goat breeders on a small-scale. Using a sterile syringe, 5 CC blood was taken by a professional technician from each participant. The samples were labeled with numbers and handled as described. The data regarding name, age, gender, clinical history, and raw milk consumption habit for each respective sample were included.

#### Serological assay

Serum samples were tested for the presence of IgG by using ID Screen^®^ Q Fever Indirect Multi-species (ID.vet innovative diagnostics, Grabels, France) following the manufacturer’s instructions. The optical densities (ODs) were measured by Stat Fax 2100 Microplate Reader (Awareness Technology INC, Fl, USA) at 450 nm. Serum positivity percentage (S/P%) was calculated as follows: S/P% = (OD tested sample − OD negative control)/(OD positive control − OD negative control) × 100. Samples with an S/P value < 40% were considered negative, those between 40 and 50% were considered inconclusive, and those between 50 and 80% were considered positive, while those gave ˃ 80% were considered strong positive.

#### Statistical analyses

Statistical analyses were performed by GraphPad Prism 5.0 software (GraphPad Software, Inc., La Jolla, CA, USA). Fisher’s exact test was used to assess the association between the categorical variables. Odds ratios (OR) with 95% confidence interval (95% CI) were computed for the data. *P* value < 0.05 was considered significant.

### Results

The prevalence and associated risk factors of *C. burnetii* IgG antibodies in sheep, goats, and humans in contact at El Minya Governorate was determined in the present study. The overall seroprevalence among small ruminants recruited to this study was 26.35% (39 of 148) with a 25.68% (28 of 109) in sheep and a 28.20% (11 of 39) in goats. Although there was no significant association between the different variables (locality, age, pregnancy status and history of abortion) and the infection rate among the examined animals, the OR indicated varied exposure rates. Additionally, no significant difference between the infection rate in small ruminants compared to humans in contact was found (Table 1).

**Table 1:** Impact of different variables on the prevalence of Q fever in small ruminants

Variable	Species examined					
	Sheep			Goat		
	No. tested	Positive no. (%)	Odds ratio (95% CI)	No. tested	Positive no. (%)	Odds ratio (95% CI)
Locality						
Dayr Mawas	42	10 (23.8)	0.85 (0.35–2.1) 1.18 (0.48–2.86)	19	3 (15.78)	0.281 (0.06–1.29) 3.56 (0.77–16.32)
Matay	67	18 (26.86)		20	8 (35)	
Total	109	28 (25.68)		39	11 (28.20)	
Age						
1–2 years	22	5 (22.73)	0.82 (0.27–2.47) 1.22 (0.4–3.69)	12	2 (16.66)	0.40 (0.07–2.23) 2.5 (0.45–13.9)
> 2 years	87	23 (26.44)		27	9 (33.33)	
Total	109	28 (25.68)		39	11 (28.20)	
Gender						
Male	–	–	–	7	1 (14.28)	0.366 (0.038–3.46) 2.73 (0.28–25.76)
Female	109	28 (25.68)	–	32	10 (31.25)	
Total	109	28 (25.68)	–	39	11 (28.20)	
Pregnancy						
Yes	71	19 (26.76)	1.2 (0.47–2.9) 0.85 (0.34–2.12)	18	6 (33.33)	1.3 (0.27–5.7) 0.8 (0.17–3.65)
No	38	9 (23.68)		14	4 (28.57)	
Total	109	28 (25.68)		32	10 (31.25)	
Abortion history						
Yes	5	2 (40)	2 (0.32–13) 0.5 (0.79–3.16)	1	1 (100)	7.1 (0.26–190.7) 0.14 (0.005–3.78)
No	104	26 (25)		31	9 (29)	
Total	109	28 (25.68)		32	10 (31.25)	

On the other hand, a 25.71% (9 of 35) humans were positive for *C. burnetii* IgG antibodies, of which 8.57% (3 of 35) were males and 17.14% (6 of 35) were females. Human gender was the only statistically significant risk factor (*P* = 0.0435) in this study (Table 2).

**Table 2:** Impact of gender and age on the prevalence of Q fever in humans

Variable	No. tested	Positive no. (%)	Negative no. (%)	Odds ratio (95% CI)
Gender				
Male	22	3 (13.64)	19 (86.36)	0.18 (0.036–0.94) 5.43 (1.058–27.84) *P* = 0.0435
Female	13	6 (46.15)	7 (53.85)	
Total	35	9 (25.71)	26 (74.29)	
Age				
15–39 years	19	4 (21.1)	15 (78.9)	0.59 (0.13–2.7) 1.7 (0.37–7.9)
40–63 years	16	5 (31.25)	11 (68.75)	
Total	35	9 (25.71)	26 (74.29)	

Two of the positive humans had a history of self-limited fever with pneumonia or hepatitis and one woman had a history of prolonged fever of unknown cause. Moreover, 3 seropositive females had a complaint of heart disorder when sampling. Finally, 5 out of the 9 seropositive individuals for Q fever were keeping seropositive animals during the sampling period (Additional file 1: Table S1).

### Discussion

As a bacterium of unique merits both outside and inside hosts [9], our information regarding *C. burnetii* and its worldwide impact on humans and animals needs expansion. Limited studies have focused on Q fever in Egypt since becoming public health concern in 1995 [10, 11]. That warrants the need for more research to realize the disease status in the previously neglected areas such as El Minya Governorate.

In the present study, the seroprevalence of *C. burnetii* IgG antibodies in El Minya Governorate was 25.68 and 28.20% in sheep and goats, respectively. Previous seroprevalence studies conducted in different Egyptian Governorates have shown somewhat comparable results. For instance, our results were higher than those reported from North Sinai, Ismailia and Qaluobia Governorates for both sheep and goats [12–14]. On the other hand, in Cairo, Giza, and El-Fayum Governorates, the Q fever seroprevalence was higher than that reported for sheep and lower for goats in the present study [15]. In other countries, Q fever seroprevalence was 23.7 and 33.9% [16, 17], respectively in Iranian sheep, 22.4% in Iranian goats [17], 8.67% in Japan [18] and 20% in Turkey [19] in sheep.

Our results showed no significant difference between the study regions. However, mixed rearing of sheep and goats in one herd at Matay district may explain the increased odds of exposure of goats to infection in that region as compared to Dayr Mawas, which does not typically house sheep and goats in one place.

As seen in our investigation, no significant relationship was observed between age and Q fever infection rate indicating that all ages were relatively at equal risk of acquiring infection. This may be due to animal exposure to a common source of infection and disease emergence in the study area [13, 16]. This result was consistent with those investigators [13, 16], meanwhile, other reports found that the age of examined sheep and goats has significantly impacted the frequency of Q fever occurrence [17, 20, 21].

Regarding gender, previous studies reported a higher seroprevalence among examined female than male animals [22–24]. Although the current study showed a similar finding with high odds of exposure among the examined female goats, the comparison is difficult because of high variation of numbers among examined animals from both genders.

In the current study, a slightly higher seroprevalence was observed among pregnant than non pregnant animals with nearly equal odds of exposure in both groups. Such a result further confirms our assumption that Q fever is emerging in the governorate and both pregnant and non-pregnant animals present a potential zoonotic risk for the humans residing in the region.

As is well-known, Q fever is mostly asymptomatic in small ruminants and abortion is the exclusive clinical consequence [3]. Keeping in mind that only three seropositive animals in the present study had a history of abortion, with the assumption that the *C. burnetii* was the definitive reason behind it, our finding also reported that most, if not all, of the seropositive animals were subclinically infected. This reinforces the fact that the vast majority of *C. burnetii* infections among animals in Egypt were inapparent because of relative tolerance of native breeds, which are commonly reared in Egypt, to infection [25].

Compared to previous studies conducted in Egypt regarding Q fever in humans, our results showed that the seroprevalence of Q fever among the tested humans in a close contact with small ruminants was 25.71% which is higher than that previously reported (23.3%) in a similar risk group [14] and those recorded by other researchers [12, 15, 25] who found that the seroprevalence of Q fever was 5, 16.3 and 19% among the individuals of intimate contact with ruminants, respectively.

Based on the current and aforementioned results, it seems clear that the study group, people in contact with ruminants, are at a growing risk of acquiring Q fever infection that warrants the need for continuous monitoring and maintaining effective source tracking. Moreover, previous Egyptian investigators have reported 72 and 32% seroprevalence rates of Q fever among human communities living in Behera Governorate and Nile River Delta of Egypt, respectively [26, 27]. In the present study, the gender was the only significant risk factor for Q fever infection in humans. Women exhibited greater odds of exposure to Q fever compared to men, which may be as a result of their active engagements in assisting parturient and aborted animals as well as drinking small ruminants’ raw milk.

Interestingly, the current study documented the presence of either historical and/or existing health complaints relevant to Q fever disease manifestations in some seropositive persons residing in the governorate. Accordingly, physicians’ awareness regarding Q fever epidemiology and clinical presentation has to be raised since prompt and precise diagnosis and intervention is strongly recommended especially in cases of *C. burnetii*-induced endocarditis [28].

Given that not all seropositive people for Q fever were rearing seroreactive animals at sampling time, effective infection source tracking remains difficult. However, in addition to the possible direct role of the examined small ruminants in transmitting Q fever to humans, the ability of *C. burnetii* to endure the drastic environmental conditions, and hence its force to persist in the environment with subsequent spread by the wind for long distances [1], also makes environment one of the possible sources of the infection. As a conclusion, the present study provides the first evidence that Q fever is circulating in animals and humans in El Minya Governorate and reinforces the fact that small ruminants are potential disease reservoirs. No statistical difference between the infection in animals and humans was found, indicating that Q fever may be emerging in the governorate. Gender was the only significant risk factor for human infection. Collectively, our study lays the foundation for early prediction and better management of possible Q fever outbreaks in the future and underscores the urgent need to initiate the perspective control measures for small ruminants and their owners.

### Limitations

Although the present study could drive both veterinary and public health authorities to commence a unified protection strategy against Q fever in the governorate, a more comprehensive epidemiological picture could be achieved by extending the survey area and incorporating more disease hosts.

## Additional file

**Additional file 1: Table S1.** Summarized data for the seropositive people to *C. burnetii.*

## Abbreviations

°C: celsius
*C. burnetii*: *Coxiella burnetii*
CC: cubic centimeter
CI: confidence interval
ELISA: enzyme-linked immunosorbent assay
IgG: immunoglobulin G
Km: kilometer
nm: nanometer
OD: optical density
OR: odds ratio
*P*: probability
“Q” fever: “query” fever
S/P%: seropositivity percentage
×*g*: times gravity
~: approximately
<: less than
>: greater than

## References

[1] Maurin M, Raoult D (1999). Q fever. Clin Microbiol Rev.

[2] Minnick MF, Raghavan R (2011). Genetics of *Coxiella burnetii*: on the path of specialization. Futur Microbiol.

[3] Van den Brom R, van Engelen E, Roest HIJ, van der Hoek W, Vellema P (2015). *Coxiella burnetii* infections in sheep or goats: an opinionated review. Vet Microbiol.

[4] Weese JS, Fulford MB, editors. Companion animal zoonoses. Oxford: Wiley-Blackwell; 2011. 10.1002/9780470958957. Accessed 27 Jul 2017.

[5] Lang GH (1989). Q fever: an emerging public health concern in Canada. Can J Vet Res.

[6] Mostafavi E, Rastad H, Khalili M (2012). Q fever: an emerging public health concern in Iran. Asian J Epidemiol.

[7] Schimmer B, Notermans DW, Harms MG, Reimerink JHJ, Bakker J, Schneeberger P (2012). Low seroprevalence of Q fever in The Netherlands prior to a series of large outbreaks. Epidemiol Infect.

[8] Bosnjak E, Hvass AMSW, Villumsen S, Nielsen H (2010). Emerging evidence for Q fever in humans in Denmark: role of contact with dairy cattle. Clin Microbiol Infect.

[9] Gürtler L, Bauerfeind U, Blümel J, Burger R, Drosten C, Gröner A (2014). *Coxiella burnetii*—pathogenic agent of Q (query) fever. Transfus Med Hemother.

[10] Botros BA, Soliman AK, Salib AW, Olson J, Hibbs RG, Williams JC (1995). *Coxiella burnetii* antibody prevalences among human populations in north-east Africa determined by enzyme immunoassay. J Trop Med Hyg.

[11] Gwida M, El-Ashker M, El-Diasty M, Engelhardt C, Khan I, Neubauer H (2014). Q fever in cattle in some Egyptian Governorates: a preliminary study. BMC Res Notes.

[12] Mazyad SAM, Hafez AO (2007). Q fever (*Coxiella burnetii*) among man and farm animals in North Sinai, Egypt. J Egypt Soc Parasitol.

[13] El-Mahallawy HS, Abou-Eisha AM, Fadel HM (2012). *Coxiella burnetii* infections among small ruminants in Ismailia Governorate. SCVMJ.

[14] Khalifa NO, Elhofy FI, Fahmy HA, Sobhy MM, Agag MA (2016). Seropervelance and molecular detection of *Coxiella burnetii* infection in sheep, goats and human in Egypt. ISOI J Microbiol Biotechnol Food Sci.

[15] Nahed HG, Khaled AA-M (2012). Seroprevalence of *Coxiella burnetii* antibodies among farm animals and human contacts in Egypt. J Am Sci.

[16] Esmaeili S, Bagheri Amiri F, Mostafavi E (2014). Seroprevalence survey of Q fever among sheep in Northwestern Iran. Vector Borne Zoonotic Dis.

[17] Ezatkhah M, Alimolaei M, Khalili M, Sharifi H (2015). Seroepidemiological study of Q fever in small ruminants from Southeast Iran. J Infect Public Health.

[18] Giangaspero M, Bonfini B, Orusa R, Savini G, Osawa T, Harasawa R (2013). Epidemiological survey for *Toxoplasma gondii*, *Chlamydia psittaci* var. ovis, *Mycobacterium paratuberculosis*, *Coxiella burnetii*, *Brucella* spp., leptospirosis and Orf virus among sheep from northern districts of Japan. J Vet Med Sci.

[19] Kennerman E, Rousset E, Gölcü E, Dufour P (2010). Seroprevalence of Q fever (coxiellosis) in sheep from the Southern Marmara Region, Turkey. Comp Immunol Microbiol Infect Dis.

[20] García-Pérez AL, Astobiza I, Barandika JF, Atxaerandio R, Hurtado A, Juste RA (2009). Short communication: investigation of *Coxiella burnetii* occurrence in dairy sheep flocks by bulk-tank milk analysis and antibody level determination. J Dairy Sci.

[21] Akbarian Z, Ziay G, Schauwers W, Noormal B, Saeed I, Qanee AH (2015). Brucellosis and *Coxiella burnetii* infection in householders and their animals in secure villages in Herat Province, Afghanistan: a cross-sectional study. PLOS Negl Trop Dis.

[22] Zahid MU, Hussain MH, Saqib M, Neubauer H, Abbas G, Khan I (2016). Seroprevalence of Q fever (Coxiellosis) in small ruminants of two districts in Punjab, Pakistan. Vector Borne Zoonotic Dis.

[23] Cetinkaya B, Kalender H, Ertas HB, Muz A, Arslan N, Ongor H (2000). Seroprevalence of coxiellosis in cattle, sheep and people in the east of Turkey. Vet Rec.

[24] Sakhaee E, Khalili M (2010). The first serologic study of Q fever in sheep in Iran. Trop Anim Health Prod.

[25] Abdel-Moein KA, Hamza DA (2017). The burden of *Coxiella burnetii* among aborted dairy animals in Egypt and its public health implications. Acta Trop.

[26] Samaha HA, Haggag YN, Nossair MA, Samar A (2012). Serological detection of IgG against *C. burnetti* phase II in Behera Province Western Egypt. Alex J Vet Sci.

[27] Corwin A, Habib M, Watts D, Darwish M, Olson J, Botros B (1993). Community-based prevalence profile of arboviral, rickettsial, and Hantaan-like viral antibody in the Nile River Delta of Egypt. Am J Trop Med Hyg.

[28] Anderson A, Bijlmer H, Fournier PE, Graves S, Hartzell JD, Kersh GJ (2013). Diagnosis and management of Q fever—United States, 2013: recommendations from CDC and the Q fever working group. Mmwr.

