# Supplementary material for: Cross-sectional study for determining the prevalence of Q fever in small ruminants and humans at El Minya Governorate, Egypt
Source: BMC Res Notes. 2017 Oct 30;10:538. doi: 10.1186/s13104-017-2868-2 (PMC5663073; doi:10.1186/s13104-017-2868-2)
Supplement: Supplementary file 1 — Additional file 1: Table S1. Summarized data for the seropositive people to C. burnetii. [file 13104_2017_2868_MOESM1_ESM.docx]

**Table S1: Summarized data for the seropositive people to *C. burnetii***

| **Patient No.** | **Gender** | **Occupation** | **Previous history** | **Complaint at time of sampling** | **Seropositive contact animals** |
| --- | --- | --- | --- | --- | --- |
| 1st | Male | Vet. worker | - | - | No |
| 2nd | Male | Farmer | - | - | Yes |
| 3rd | Male | Farmer | - | - | Yes |
| 4th | Female | Housewife | Self-limited fever + pneumonia | Heart disorder | No |
| 5th | Female | Housewife | Self-limited fever + hepatitis | Heart disorder | Yes |
| 6th | Female | Housewife | Prolonged fever of unknown cause | - | No |
| 7th | Female | Housewife | - | - | Yes |
| 8th | Female | Housewife | - | Heart disorder | Yes |
| 9th | Female | Housewife | - | - | No |
